# The association between serotonin-related gene polymorphisms and susceptibility and early sertraline response in patients with panic disorder

**DOI:** 10.1186/s12888-020-02790-y

**Published:** 2020-07-28

**Authors:** Zhili Zou, Yulan Huang, Jinyu Wang, Wenjiao Min, Bo Zhou

**Affiliations:** Psychosomatic Department, Sichuan Academy of Medical Science & Sichuan Provincial People’s Hospital, No. 32 West Second Section First Ring Road, Chengdu, Sichuan 610072 P.R. China

**Keywords:** Panic disorder, Serotonin transporter, Polymorphism, Setraline

## Abstract

**Background:**

A number of studies have shown that genetic factor plays an important role in etiology of panic disorder (PD). The aim of the present study was to examine the association of serotonin-related gene polymorphisms with PD risk. Then, we analyzed the correlation between these gene polymorphisms and response to sertraline drug.

**Methods:**

Two hundred thirty-three patients with PD and 231 healthy controls were enrolled in the study. Panic Disorder Severity Scale (PDSS) were administered to all subjects, and all patients in the study were also assessed after 4 weeks of treatment. The SLC6A4(rs140701, rs3813034, 5-HTTLPR and STin2), 5-HTR1A rs6295, 5-HTR2A rs6313 and COMT rs4680 gene polymorphisms were genotyped and assessed for the potential association.

**Results:**

The allelic model showed that the SLC6A4 rs140701 polymorphism variant was significantly associated with increased risk of PD (OR = 0.624, 95% CI 0.450–0.864, *p* < 0.05), and a significant result was found in the dominant model (OR = 0.546; 95% CI, 0.371–0.804, *p* < 0.05). There was a significant difference in allele and genotype frequency between responders and nonresponders in the 5-HTTLPR polymorphism (OR = 0.205, 95% CI 0.128–0.328; OR = 0.249, 95% CI 0.155–0.401, both *p* < 0.001), indicating the PD patients with S-allele had a poorer response to sertraline than L-allele carriers.

**Conclusions:**

The present study suggests that the SLC6A4 rs140701 polymorphism variant may be associated with susceptibility to PD, and 5-HTTLPR polymorphism may be a predictor of response to sertraline in the treatment of PD.

## Background

Panic disorder is a common anxiety disorder characterized by sudden and unexpected panic attacks and accompany with obviously anticipatory anxiety. The estimated lifetime prevalence of PD is 3.4 to 4.7% [1, 2]. It typically occurs in young adults, and women are more likely to be affected than men [3]. However, the etiology of PD is complex and unclear. A meta-analysis showed genetic factors explain approximately 43% of the variance in the PD [4], indicating an important role in the pathological PD. Previous studies have consistently demonstrated serotonin involvement in the neurobiology of PD [5, 6]. Clinical studies also demonstrated that selective serotonin-reuptake inhibitors (SSRIs) increasing the synaptic availability of 5-HT and are effective in the treatment of PD [7]. Therefore, the certain variants in the serotonin-related genes, such as serotonin transporter (5-HTT), 5-HT1A receptor (5-HTR1A), 5-HT2A receptor (5HTR2A) and catechol-O-methyltransferase (COMT) genes, may influence 5-HT neurotransmission, and they are good candidates for the study of PD. A few studies have investigated the association between the 5-HTT linked polymorphic region (5-HTTLPR), 5-HTR1A rs6295, 5-HTR2A rs6313, COMT rs4680 polymorphisms and PD [8–16]. However, the results were hardly replicable and the pathogenesis of PD remains to be clarified. It is important to note that previous studies have been conducted in different ethnic groups, few of them have examined the relationship between these gene polymorphisms and PD in Chinese population.

SSRIs are recommended as the first-line antidepressants in treatment for PD. However, not all patients can gain full response from these antidepressants. With the advancement of pharmacogenomic technologies, genetic variation has been identified to contribute to individual response to antidepressants. Pharmacogenomic studies focusing on candidate gene polymorphisms implicated in antidepressant response, especially in the serotonergic pathway. Abundant evidence has shown that serotonin-related gene polymorphisms played an important role in the clinical effects of antidepressants. For example, a few studies investigated that 5-HTTLPR, intron 2 (STin2), 5-HTR1A rs6295, 5-HTR2A rs6313 and COMT rs4680 polymorphisms associated with antidepressant response [17–26]. However, these findings are inconsistent and do not completely explain the predictive effects of antidepressants. To our knowledge, many previous studies have been conducted in other psychiatric disorders, such as major depressive disorder(MDD) [27–30], and few of them have conducted in PD. In addition, the different results of these studies might also be partly attributable to the differences in the type of antidepressant drug [31, 32]. Sertraline, a SSRI which has been used widely to treat PD for its prominent therapeutical effects and low incidence of adverse events [33], was the first-line treatment for PD. Hence, the first aim of this study was to investigate the association of serotonin-related gene polymorphisms including SLC6A4 (rs140701, rs3813034, 5-HTTLPR and STin2), 5-HTR1A rs6295, 5-HTR2A rs6313 and COMT rs4680 with PD risk, and the second aim was to examine the association between these single nucleotide polymorphisms (SNPs) and early response to sertraline treatment in PD.

## Methods

### Participants

Two hundred thirty-three patients with PD were recruited from inpatient and outpatient populations in the Department of Psychosomatic, Sichuan Provincial People’s Hospital between May 2015 and December 2018(recruitment flow chart in Fig. S1). Diagnosis of PD was conducted according to the Structured Clinical Interview for the Diagnostic and Statistical Manual of Mental Disorders fourth edition (DSM-IV) (SCID-1) [34], which was administered by trained clinical psychiatrists. Patients with neurological diseases, and/or past or current episodes of MDD, generalized anxiety disorder(GAD), manic disorder, bipolar disorder, schizophrenia or any other psychiatric disorders were excluded. In addition, 231 healthy controls were recruited from the Center of Health Examination, Sichuan Provincial People’s Hospital. The SCID-1 was also performed to exclude a lifetime or current diagnosis of PD, MDD, GAD, among others.

All subjects in this study were Han Chinese, and all subjects were free of acute or chronic somatic disorders. All patients were free of antidepressants or other psychotropic medications intake within 2 weeks before their examination. Demographic data and clinical presentations were obtained from medical records or qualified interviews. A 3-mL EDTA-anticoagulated peripheral blood sample was collected from every individual.

### Measures

#### Panic disorder severity scale(PDSS)

The Panic Disorder Severity Scale is a valid and reliable inventory. Seven items are included in this scale, and each item is scored 0–4 on a Likert scale ranging from “none” to “extremely severe” [35]. Also, the scale was translated into Chinese, and the PDSS-Chinese Version(PDSS-CV) has good internal consistency (Cronbach’s alpha) with the overall score (0.83) [36].

#### Treatment

Setraline (100-200 mg qd) was administered to all patients for 4-week period. Other psychotropic medications were not permitted during the study except for benzodiazepine, which was prescribed occasionally for insomnia with a minimal dosage at bedtime. PDSS was assessed to all patients at baseline and after 4 weeks of treatment. Response was defined as a 40% or greater reduction in scores on the pre-treatment PDSS [37].

#### SNP selection and genotyping

The SNP selected was based on literature search and minor allele frequency(MAF) > 0.05 of east Asian population from public databases. The references were listed in the Table S1. SNP genotyping was performed using an improved multiplex ligation detection reaction (iMLDR) technique which developed by Genesky Biotechnologies Inc. (Shanghai, China). The primer information of the reaction mixtures is described in Tables S2 and S3. The 20 μl multiplex polymerase chain reaction (PCR) included 1 x GC-I buffer (Takara), 0.3 mM dNTP, 3.0 mM Mg^2+^, 1 U HotStar Taq Polymerase (Qiagen Inc), 1 μl genomic DNA(5-10 ng/μl), and 1 μl Multiplex-PCR primermix. In addition, The 5-HTT of PCR reaction volume(10 μl) included 10 x buffer I (Qiagen Inc), Q Solution(Qiagen Inc.), 0.2 mM Mg 2+, 1 U HotStarTaq polymerase (Qiagen Inc.), 1 μl genomic DNA, and 1 μl PCR primermix. The PCR cycling program was as follows: denaturation at 95 °C for 2 min, followed by 11 cycles of 94 °C for 20s, annealing at 65 °C for 40 s and 72 °C for 1.5 min, and each cycle decreased 0.5 °C. The third step was set as 24 cycles of 94 °C for 20s, 59 °C for 30 s, finally, 72 °C for 2 min and 4 °C for hold. In addition, the 5-HTT of cycling program for PCR was 95 °C for 2 min, 35 cycles of amplification consisted of 94 °C for 20 s and 72 °C for 1.5 min, and final extension at 68 °C for 60 min and a hold at 4 °C. 5 U SAP and 2 ExonucleaseIwere used to purify the PCR product at 37 °C and then 15 min of 75 °C for inactivation. 2 μl purified multiplex PCR product, together with 6 μl ddH_2_O, were added into the ligation reaction containing 1 μl of 10× ligation buffer, 0.25 μl Tag DNA ligase, 5′ ligationprimer (1 μM) 0.4 μl, 3′ ligationprimer (2 μM) 0.4 μl. The ligation cycling program was set as 38 cycles× (94 °C for 1.5 min and 56 °C for 4 min), and a hold at 4 °C. After PCR, 0.5 μl ligation product was taken for Sequencing with 0.5 μl Liz500 Size Standard, and 9 μl HiDi mixture (ABI3730XL). The raw data were analyzed by Gene Mapper v4.1 software (AppliedBiosystems, USA). All primers, probes and labeling oligos were designed by and ordered from Genesky Biotechnologies Inc.

### Statistical analysis

The data were analyzed using SPSS version 18.0 software (SPSS Inc., Chicago, IL, USA). Student’s *t*-tests were used for inter group comparisons of continuous variables, with Pearson’s chi-square tests utilized for categorical variables. *P* values for the Hardy-Weinberg equilibrium (HWE) were tested by Pearson’s chi-square test, and *P* > 0.05 indicated no significant deviation in allele and genotype frequencies among subjects. Associations between SNPs and disease status were determined based on the distributions of allelic frequencies and genetic models (additive, dominant, and recessive model), and odds ratios (*ORs*) and 95% confidence intervals (*CIs*) were performed in unconditional logistic regression analysis using PLINK v1.07, 20 adjusting for age, gender, educational level and resident location. To protect from Type I error, a Bonferroni correction was conducted. For all analyses, statistical tests were two-tailed and an alpha level of 0.05 was used to define statistical significance. Power calculation (α = 0.05) was performed using Power and Sample Size Calculation Software (3.6.1).

## Results

### Demographic data and clinical manifestations

A total of 233 patients with PD (92 men, 141 women) and 231 controls (98 men, 133 women) were selected. The average age of the study sample was (35.65 ± 9.77) years, and the mean age of the control group was (36.96 ± 7.82) years. 53.6% of the patients (*n* = 125) were residing in urban locations, and 46.4% of the patients (*n* = 108) were residing in rural locations. No statistically significant differences were noted between cases and controls in terms of sex, age, education level and resident location(*p* > 0.05). In addition, the mean total duration of panic disorder was (2.49 ± 1.45) years. The PDSS score was 15.47 ± 3.82 before medication and 9.51 ± 3.81 after 4 weeks of medication, and the score was significantly reduced after medication (*p* < 0.05). According to the definition of remission, the response rate was 42.1%(*n* = 98). The demographic details of the sample are given in Table 1.

**Table 1:** Demographic and clinical characteristics in patients with PD and controls

Variable	PD (*n* = 233)	Controls(*n* = 231)	t/χ 2-value	*p*-value
**Sex, n (%)**				
Female	141(60.5)	133 (57.6)	0.414	0.520
Male	92 (39.5)	98 (42.4)		
**Age**	35.65 ± 9.77	36.96 ± 7.82	1.594	0.112
**Educational level, n (%)**				
< Junior high school	49 (21.0)	42 (18.2)		
High school	95 (40.8)	80 (34.6)	3.836	0.147
College and above	89 (38.2)	109 (47.2)		
**Resident location, n (%)**				
Urban	125 (53.6)	129 (55.8)		
Rural	108 (46.4)	102 (44.2)	0.266	0.635
Total duration of PD, years	2.49 ± 1.45			
PDSS baseline	15.47 ± 3.82			
PDSS 4-week	9.51 ± 3.81			

### Association of serotonin-related gene polymorphisms with PD risk

To determine whether the sample size of this study has power to detect the real difference, a power analysis was performed. From the result of Table S4, the sample size of 233 vs 231 ensured that a two-sided test with α = 0.05 had 80% power to detect an relative risk of 2.0 (Ψ = 2.0). All selected SNPs fulfilled the HWE in both cases and controls(*p* > 0.05). To determine the extent of linkage disequilibrium (LD) between the two polymorphisms (rs140701, rs3813034) in SLC6A4 gene, standardized LD coefficient D’ was calculated for this pair of polymorphisms. The R^2^ and D’ was 0.510 and 0.717 respectively, indicating weak LD between these two polymorphisms.

The allele distributions of SLC6A4 (rs140701 and rs3813034) were significantly different between PD cases and the controls (OR = 0.624, 95% CI 0.450–0.864, *p* = 0.004; OR = 0.705, 95% CI 0.509–0.975, *p* = 0.034; respectively). However, only SLC6A4 rs140701 remained significant after adjusting for Bonferroni’s multiple testing(*p* = 0.028). After adjustment for age, gender, educational level and resident location, the dominant model of SLC6A4 rs140701 showed the relative risk of PD for genotype CC + CT was lower than for genotype TT (OR = 0.546; 95% CI, 0.371–0.804, *p* = 0.004), and after Bonferroni correction for multiple comparisons, the significance remained (*p* = 0.028).

In addition, the dominant model of rs3813034 showed the relative risk of PD for genotype CA + AA was lower than for genotype CC (OR = 0.636; 95% CI, 0.432–0.935, *p* = 0.025). For 5-HTTLPR polymorphism, the relative risk of PD for genotype LL + LS was lower than for genotype SS (OR = 0.632; 95% CI, 0.436–0.916; *p* = 0.021). The additive model of rs140701 and rs3813034 showed similar association with PD(OR = 0.638, 95% CI 0.463–0.879, *p* = 0.009; OR = 0.721, 95% CI 0.526–0.988, *p* = 0.048; respectively). However, these correlations were no longer significant after adjusting for Bonferroni’s multiple testing (*p* > 0.05). Also, there were no significant associations of other SNPs with PD in allelic or other models (*p* > 0.05) (Table 2).

**Table 2:** Association of serotonin-related gene polymorphisms with PD risk in Chinese Han population

Gene	SNP	Alleles and Genotypes (n, %)	PD (*n* = 233)	Controls (*n* = 231)	Model	OR (95% CI)	*p*-value	*p*-value^corr^
SLC6A4	rs140701	C	76 (16.3)	110 (23.8)	Allele^a^	0.624 (0.450–0.864)	0.004	0.028
		T	390 (83.7)	352 (76.2)				
		CC	10 (4.3)	13 (5.6)	Additive^b^	0.638 (0.463–0.879)	0.009	0.063
		CT	56 (24.0)	84 (36.4)	Dominant^b^	0.546 (0.371–0.804)	0.004	0.028
		TT	167 (71.7)	134 (58.0)	Recessive^b^	0.752 (0.323–1.751)	0.438	1
	rs3813034	C	386 (82.8)	357 (77.3)	Allele^a^	0.705 (0.509–0.975)	0.034	0.238
		A	80 (17.2)	105 (22.7)				
		CC	164 (70.4)	139 (60.2)	Additive^b^	0.721 (0.526–0.988)	0.048	0.336
		CA	58 (24.9)	79 (34.2)	Dominant^b^	0.636 (0.432–0.935)	0.025	0.175
		AA	11 (4.7)	13 (5.6)	Recessive^b^	0.831 (0.364–1.895)	0.662	1
	5-HTTLPR	L	107 (23.0)	130 (28.1)	Allele^a^	0.761 (0.566–1.024)	0.071	0.497
		S	359 (77.0)	332 (71.9)				
		LL	22 (9.4)	20 (8.7)	Additive^b^	0.785 (0.594–1.039)	0.100	0.700
		LS	63 (27.0)	90 (39.0)	Dominant^b^	0.632 (0.436–0.916)	0.021	0.147
		SS	148 (63.5)	121 (52.3)	Recessive^b^	1.100 (0.583–2.076)	0.842	1
	STin2	10	44 (9.4)	43 (9.3)	Allele^a^	1.016 (0.653–1.580)	0.944	1
		12	422 (90.6)	419 (90.7)				
		10/10	5 (2.1)	2 (0.9)	Additive^b^	1.015 (0.663–1.554)	0.956	1
		12/10	34 (14.6)	39 (16.9)	Dominant^b^	0.932 (0.575–1.508)	0.711	1
		12/12	194 (83.3)	190 (82.2)	Recessive^b^	2.511 (0.482–13.080)	0.393	1
HTRA1	rs6295	G	359 (77.0)	358 (77.5)	Allele^a^	1.026 (0.755–1.395)	0.870	1
		C	107 (23.0)	104 (22.5)				
		GG	138 (59.2)	138 (59.7)	Additive^b^	1.026 (0.754–1.398)	0.554	1
		GC	83 (35.6)	82 (35.5)	Dominant^b^	1.022 (0.705–1.480)	0.560	1
		CC	12 (5.2)	11 (4.8)	Recessive^b^	1.086 (0.469–2.513)	0.772	1
HTR2A	rs6313	G	163 (35.0)	179 (38.7)	Allele^a^	0.868 (0.651–1.111)	0.234	1
		A	303 (65.0)	283 (61.3)				
		AA	106 (45.5)	86 (37.2)	Additive^b^	0.859 (0.663–1.113)	0.252	1
		GA	91 (39.1)	111 (48.1)	Dominant^b^	0.711 (0.490–1.030)	0.061	0.427
		GG	36 (15.4)	34 (14.7)	Recessive^b^	1.059 (0.637–1.761)	0.749	1
COMT	rs4680	A	143 (30.7)	118 (25.5)	Allele^a^	1.291 (0.969–1.720)	0.081	0.567
		G	323 (69.3)	344 (74.5)				
		AA	24 (10.3)	15 (6.5)	Additive^b^	1.284 (0.65–1.707)	0.118	0.826
		GA	95 (40.8)	88 (38.1)	Dominant^b^	1.297 (0.901–1.869)	0.175	1
		GG	114 (48.9)	128 (55.4)	Recessive^b^	1.654 (0.844–3.240)	0.236	1

“A” represent wild type and “a” represent mutant type: allele, a vs. A; additive, aa vs. Aa vs. AA; dominant, aa + Aa vs. AA, recessive, aa vs. Aa +AA. ^a^ Chi-square test, ^b^ Logistic regression analyses by adjustment for age, gender, educational level and resident location. *P*^corr^: adjusted by boferroni multiple comparison correction

### Association between serotonin related gene polymorphisms and early response to sertralines in the treatment of PD

Finally, we investigated whether variations of gene could predict response to sertraline in Han Chinese population with PD. There was a significant difference in allele and genotype frequency between responders and nonresponders in the 5-HTTLPR polymorphism (OR = 0.205, 95% CI 0.128–0.328; OR = 0.249, 95% CI 0.155–0.401, all *p*^corr^ = 0.000; respectively), and the dominant and recessive model of 5-HTTLPR showed significant association with therapeutic response, indicating the PD patients with S-allele had a poorer response to sertraline than L-allele carriers. However, genotype and allele frequencies of the other gene polymorphisms were not significantly different between responders and nonresponders (*p* > 0.05). (Table 3).

**Table 3:** Genotype and allele frequencies of serotonin-related gene polymorphisms between responder and nonresponder

Gene	SNP	Alleles and Genotypes (n, %)	Responder (*n* = 98)	Nonresponder (*n* = 135)	Model	OR (95% CI)	*p*-value	*p*-value^corr^
SLC6A4	rs140701	C	37 (18.9)	39 (14.4)	Allele^a^	0.726 (0.443–1.188)	0.201	1
		T	159 (81.1)	231 (85.6)				
		CC	5 (5.1)	5 (3.7)	Additive^b^	0.638 (0.470–1.198)	0.159	1
		CT	27 (27.6)	29 (21.5)	Dominant^b^	0.694 (0.391–1.232)	0.160	1
		TT	66 (67.3)	101 (74.8)	Recessive^b^	0.715 (0.201–2.542)	0.446	1
	rs3813034	C	155 (79.1)	231 (85.6)	Allele^a^	0.638 (0.394–1.035)	0.067	0.469
		A	41 (20.9)	39 (14.4)				
		CC	63 (64.3)	101 (74.8)	Additive^b^	0.669 (0.423–1.060)	0.113	0.791
		CA	29 (29.6)	29 (21.5)	Dominant^b^	0.606 (0.344–1.069)	0.128	0.896
		AA	6 (6.1)	5 (3.7)	Recessive^b^	0.590 (0.175–1.991)	0.332	1
	5-HTTLPR	L	76 (38.8)	31 (11.5)	Allele^a^	0.205 (0.128–0.328)	0.000	0.000
		S	120 (61.2)	239 (88.5)				
		LL	16 (16.3)	6 (4.4)	Additive^b^	0.249 (0.155–0.401)	0.000	0.000
		LS	44 (44.9)	19 (14.1)	Dominant^b^	0.144 (0.079–0.261)	0.000	0.000
		SS	38 (38.8)	110 (81.5)	Recessive^b^	0.238 (0.090–0.634)	0.004	0.029
	STin2	10	22 (11.2)	22 (8.1)	Allele^a^	0.702 (0.377–1.307	0.262	1
		12	174 (88.8)	248 (91.9)				
		10/10	3 (3.1)	2 (1.5)	Additive^b^	0.734 (0.410–1.315)	0.372	1
		12/10	16 (16.3)	18 (13.3)	Dominant^b^	0.723 (0.363–1.442)	0.454	1
		12/12	79 (80.6)	115 (85.2)	Recessive^b^	0.476 (0.078–2.905)	0.428	1
HTRA1	rs6295	G	151 (77.0)	208 (77.0)	Allele^a^	1.000 (0.646–1.549)	0.999	1
		C	45 (23.0)	62 (23.0)				
		GG	59 (60.2)	79 (58.5)	Additive^b^	1.000 (0.645–1.551)	0.985	1
		GC	33 (33.7)	50 (37.0)	Dominant^b^	1.072 (0.631–1.822)	0.903	1
		CC	6 (6.1)	6 (4.4)	Recessive^b^	0.713 (0.223–2.281)	0.826	1
HTR2A	rs6313	G	62 (31.6)	101 (37.4)	Allele^a^	1.292 (0.875–1.906)	0.197	1
		A	134 (68.4)	169 (62.6)				
		AA	48 (49.0)	58 (43.0)	Additive^b^	1.252 (0.869–1.805)	0.199	1
		GA	38 (38.8)	53 (39.3)	Dominant^b^	1.274 (0.756–2.149)	0.233	1
		GG	12 (12.2)	24 (17.8)	Recessive^b^	1.550 (0.733–3.274)	0.355	1
COMT	rs4680	A	66 (33.7)	77 (28.5)	Allele^a^	0.786 (0.528–1.169)	0.234	1
		G	130 (66.3)	193 (71.5)				
		AA	11 (11.2)	13 (9.6)	Additive^b^	0.793 (0.537–1.172)	0.294	1
		GA	44 (44.9)	51 (37.8)	Dominant^b^	0.705 (0.418–1.189)	0.174	1
		GG	43 (43.9)	71 (52.6)	Recessive^b^	0.843 (0.361–1.969)	0.954	1

“A” represent wild type and “a” represent mutant type: allele, a vs. A; additive, aa vs. Aa vs. AA; dominant, aa + Aa vs. AA, recessive, aa vs. Aa +AA. ^a^ Chi-square test, ^b^ Logistic regression analyses by adjustment for age, gender, educational level and resident location. *P*^corr^: adjusted by boferroni multiple comparison correction

## Discussion

In this present study, a significant relationship was found between the SLC6A4 rs140701 polymorphism and PD. The patients with PD possessed significantly higher frequencies of the TT genotype compared to the controls. This result is consistent with the previous study reported that only SNP rs140701 was associated with PD in a 163 sample of African American [9]. Similar studies have found a nominally significant association of rs140701 with social anxiety disorder [38]. The 5-HTT is encoded by the solute carrier family 6 member 4 (SLC6A4) gene, at locus 17q11.2. SNP rs140701 is located in intron 9 of the serotonin transporter gene. SLC6A4 is involved in the transport of serotonin from synaptic spaces to presynaptic neurons, and the pool of available serotonin for subsequent release [39]. Collective evidence suggests the involvement of the serotonin system in the neurobiology and pharmacotherapy of PD [5–7]. Specific SLC6A4 variants may be associated with PD. In addition, previous studies have found 5-HTT knockout mice showed increased anxiety-like behaviour. Such cases have been shown in an animal experiment which found homozygous 5-HTT knockout mice were more anxious [40, 41], while 5-HTT overexpressing mice displayed reduced anxiety-like behaviour [42]. These findings suggest that the importance of the SLC6A4 gene polymorphisms in genetic association studies of PD. However, the SNP rs140701 of function is unclear and remains to be clarified in future studies.

However, we did not find any significant association evidence within the 5-HTTLPR, rs3813034 and STin2 polymorphisms of the SLC6A4 gene, which is consistent with findings from other studies and meta-analysis demonstrating that the 5-HTTLPR and STin2 were not associated with PD [43, 44]. However, inconsistent with previous research which has found the rs3813034 of SLC6A4 is a putative risk factor for PD and other behavioral disorders that involve dysregulation of serotonergic neurotransmission [45]. Additionally, we found no significant statistical differences in the genotype distributions or allele frequencies of the SNPs (5-HTR1A rs6295, 5-HTR2A rs6313 and COMT rs4680) between PD and control groups. In a study comprised of 119 PD patients and 119 healthy controls from Japanese population, no significant differences were found in the allele frequencies or genotype distributions of the COMT rs4680 or the 5-HT1A (rs6295) between PD patients and controls [10], but other studies have found significant relationship between the COMT Val158Met (rs4680) polymorphism and PD [14, 15]. In a meta analysis, the COMT Val158Met polymorphism has been found to be associated with PD in European ancestry, but not Asian ancestry [46]. Previous studies also found pure PD was associated with HTR2A 102 T-C (rs6313) polymorphism [47], but not in other study [14]. The above findings of genetic association studies are limited and inconsistent. These mixed results of studies might be attributable to different samples, sex, races, agoraphobia co-morbidity or severity of PD. For example, previous study showed that the HTR2A rs6311 polymorphism was associated with the severity of PD [43]. Above all, these conflicting findings indicate that the contribution of genetic factors to PD may involve a complex network of variants.

In the present study, we only found significant correlation between 5-HTTLPR polymorphism and treatment response to sertraline after 4-weeks treatment for PD. The results of our study indicated the PD patients with S-allele was linked to poorer response to sertraline during the early stage of treatment, suggesting that 5-HTTLPR could be a predictor of response to sertraline treatment. 5-HTTLPR is a 44 base pair insertion–deletion polymorphism which can exist as a long (L) variant of a 16 repeat sequence or a short (S) variant of 14 repeats [48]. Previous researches indicated the L allele was associated with higher levels of transcription and concentrations of 5-HTT mRNA compared to the S allele, and the short (S) allele of the 5-HTTLPR polymorphism resulted in less efficient transcription [49–51]. This may explain the fact to some extent that the SSRIs are effective in the treatment of PD with the SLC6A4 being the primary drug target. These findings may contribute to our understanding of the association between 5-HTTLPR polymorphism and antidepressant response. Consistent study found the 5-HTTLPR low-expression genotypes showed a more favorable response to exposure-based behavior therapy in PD with agoraphobia [52]. Similar findings were found in subjects with depressive disorder treated with sertraline [53]. However, previous report found no association of the 5-HTTLPR polymorphism with treatment response in a sample size of 102 PD patients treated with sertraline or paroxetine [54]. Also, there was no significant associations between the 5-HTTLPR polymorphism and sertraline responses in MDD patients [55]. On the one hand, other polymorphisms of SLC6A4 may have a combined effect with 5-HTTLPR. In addtion, the variants of cytochrome P450 enzyme genes may also contribute to the pharmacokinetics. One study found the CYP2C19 genetic polymorphism is associated with Escitalopram treatment response in Chinese patients with PD [56]. On the other hand, functional variants in SLC6A4 may be related to epigenetic mechanisms and affect gene expression. For instance, epigenetic modifications of SLC6A4 gene is showing promising results as biomarkers for prediction of antidepressant response [57]. Notably, evidence has been shown that different ethnicities responded differently to antidepressants possibly due to the variants in the SLC6A4 gene. One Meta-analysis showed 5-HTTLPR may be a predictor of antidepressant response and remission in Caucasians, while it does not appear to play a major role in Asians [58]. Last but not the least, we did not find the relationship between other gene polymorphisms and treatment response to sertraline. This is inconsistent with previous studies that found the 5-HT1A receptor -1019C/G polymorphism was strongly associated with response to treatment in PD receiving sertraline or paroxetinethe [54, 59]. Another study suggested that the genetic variant of the COMT enzyme may be related to treatment response to paroxetine in PD [60]. Inconsistent results may be related to small sample size, short follow-up periods, definition of response, antidepressant choice and ethnic differences. In addition, gene-environment interactions may also contribute to these inconsistent findings and provide more robust predictors of treatment response.

## Conclusions

The present study suggests that the SLC6A4 rs140701 variant may be associated with susceptibility to PD, and 5-HTTLPR polymorphism may be a predictor of response to sertralines in the treatment of PD. However, the results of our study should be considered preliminary in light of several limitations: (1) The sample size was limited and only from Sichuan provinces of western China, which may not completely represent the Chinese ethnicity. (2) The etiology of PD is complex and comprises environmental and genetic factors, a potential gene-environment interaction or epigenetics study should be investigated. (3) We did not measure the relationship between plasma sertraline concentration and clinical response.

## Supplementary information

**Additional file 1: Table S1.** Characteristics of circadian genes and SNPs in this study

**Additional file 2: Table S2.** The primer sequence in PCR mixture

**Additional file 3: Table S3.** the probe sequence in probe mixture

**Additional file 4: Table S4.** Power of the study at current sample size

**Additional file 5: Figure S1.** PD patients recruit flow chart.

## Data Availability

The datasets used and analyzed during the current study are available from the corresponding author on reasonable request.

## Abbreviations

COMT: Catechol-O-Methyltransferase
CIs: Confidence Intervals
CYP450: Cytochrome P450
DSM-IV: Diagnostic and Statistical Manual of Mental Disorders fourth edition
HWE: Hardy-Weinberg Equilibrium
L: Long
LD: linkage disequilibrium
MDD: Major Depressive Disorder
ORs: Odds Ratios
PD: Panic disorder
PDSS: Panic Disorder Severity Scale
PCR: Polymerase Chain Reaction
SCID: Structured Clinical Interview for DSM-IV
SNPs: Single Nucleotide Polymorphism
S: Short
SLC6A4: Solute Carrier Family 6 Member 4
SSRIs: Selective Serotonin-Reuptake Inhibitors
STin2: intron 2
5-HTR1A: 1A receptor
5-HTR2A: 5-Hydroxytryptamine 2A receptor
5-HTT: Serotonin Transporter
5-HTTLPR: Serotonin-Transporter-Linked Polymorphic Region
