# Supplementary material for: The association between serotonin-related gene polymorphisms and susceptibility and early sertraline response in patients with panic disorder
Source: BMC Psychiatry. 2020 Jul 28;20:388. doi: 10.1186/s12888-020-02790-y (PMC7388522; doi:10.1186/s12888-020-02790-y)
Supplement: Supplementary file 1 — Additional file 1: Table S1. Characteristics of circadian genes and SNPs in this study [file 12888_2020_2790_MOESM1_ESM.docx]

Table S1. Characteristics of circadian genes and SNPs in this study

| SNP | Ref | Alt | Gene | Related phenotype | MAF in East Asian**^*^** |
| --- | --- | --- | --- | --- | --- |
| rs140701 | C | T | SLC6A4 | PD (Panic disorder) and SAD (Social anxiety disorder) in European Americans ([PMID 18663369](https://www.ncbi.nlm.nih.gov/pubmed/18663369?dopt=Abstract)) | 0.50 |
| rs3813034 | C | A | SLC6A4 | PD in African American, European American, Hispanic American (PMID: 19969287) | 0.18 |
| 5-HTTLPR | L | S | SLC6A4 | major depressive disorder (PMID 19272758)  Obsessive compulsive disorder (OCD) (PMID 17375136) and  SAD (PMID 18629430) | 0.13 |
| STin2 | 10 | 12 | SLC6A4 | OCD (PMID 18191318) | 0.15 |
| rs6295 | G | C | HTRA1 | Major depression disorder (MDD) (PMID 19358877) and  Short-term response of anti-depressants in (PMID 19560507) | 0.20 |
| rs6313 | G | A | HTR2A | MDD (PMID 19590397) and anti-depressants response (PMID 16642436) | 0.41 |
| rs4680 | A | G | COMT | MDD ([PMID 20071037](https://www.ncbi.nlm.nih.gov/pubmed/20071037?dopt=Abstract)) and anti-depressant paroxetine response (PMID 18989660) | 0.32 |

**^*^**: Data are from 1000Genome website (<https://www.internationalgenome.org/>). PMID: PubMed Unique Identifier.
