# Supplementary material for: The association between serotonin-related gene polymorphisms and susceptibility and early sertraline response in patients with panic disorder
Source: BMC Psychiatry. 2020 Jul 28;20:388. doi: 10.1186/s12888-020-02790-y (PMC7388522; doi:10.1186/s12888-020-02790-y)
Supplement: Supplementary file 2 — Additional file 2: Table S2. The primer sequence in PCR mixture [file 12888_2020_2790_MOESM2_ESM.docx]

Table S2. The primer sequence in PCR mixture

| Primer Name | Primer Sequence |
| --- | --- |
| rs140701F | GAAGAGGAGGCTGGGACCTGAG |
| rs140701R | CTGTTACCCACCCCCAGGGTTA |
| rs3813034F | TGGGTGGCAGAGCATGTTGTAG |
| rs3813034R | CCATGCAAGCTTGTGAGTCTGTG |
| 5-HTTLPR F | CGGGATGCGGGGGAATACTGGT |
| 5-HTTLPR R | TTGCCGCTCTGAATGCCAGCAC |
| STin2 F | GCCTGGCGAGATTTGACTTTTC |
| STin2 R | GGCTGCGAGTAGAGGCTGTGAC |
| rs6295F | GGAGCCTGAATGGGAAGGTGAA |
| rs6295R | CGCGAGAACGGAGGTAGCTTTT |
| rs6313F | GAGAGGCACCCTTCACAGGAAAG |
| rs6313R | ACACCAGCCTCAGTGTTACAGAGTG |
| rs4680F | ATCGAGATCAACCCCGACTGTG |
| rs4680R | GGGCCTGGTGATAGTGGGTTTT |
