# Supplementary material for: The association between serotonin-related gene polymorphisms and susceptibility and early sertraline response in patients with panic disorder
Source: BMC Psychiatry. 2020 Jul 28;20:388. doi: 10.1186/s12888-020-02790-y (PMC7388522; doi:10.1186/s12888-020-02790-y)
Supplement: Supplementary file 3 — Additional file 3: Table S3. the probe sequence in probe mixture [file 12888_2020_2790_MOESM3_ESM.docx]

Table S3. the probe sequence in probe mixture

| Probe Name | Target Allele | Probe Sequence (5' phosphorated) |
| --- | --- | --- |
| rs140701FC | C | TTCCGCGTTCGGACTGATATGAGGAAAACTCAGCCACAACAACAGTGAC |
| rs140701FT | T | TACGGTTATTCGGGCTCCTGTGAGGAAAACTCAGCCACAACAACAGTGAT |
| rs140701FP |  | AATTCTCATCACAAGACCTTATGTGTGATTTTTTTT |
| rs3813034FA | A | TACGGTTATTCGGGCTCCTGTCTCCATACACAATTGAGTTGGTAGAATTTGTGAA |
| rs3813034FC | C | TTCCGCGTTCGGACTGATATCTCCATACACAATTGAGTTGGTAGAATTTGTGAC |
| rs3813034FP |  | TGTAAGAAAAATTTGGGGAATTCATATATTTGTT |
| rs6295FC | C | TGTTCGTGGGCCGGATTAGTTGGAAGAAGACCGAGTGTGTCTACC |
| rs6295FG | G | TCTCTCGGGTCAATTCGTCCTTTGGAAGAAGACCGAGTGTGTCTACG |
| rs6295FP |  | TTTTTAAAAAGCTACCTCCGTTCtcgTTTTTTTTTTT |
| rs6313FA | A | TGTTCGTGGGCCGGATTAGTAAATGCATCAGAAGTGTTAGCTTCTGCA |
| rs6313FG | G | TCTCTCGGGTCAATTCGTCCTTAAATGCATCAGAAGTGTTAGCTTCTACG |
| rs6313FP |  | GAGTTAAAGTCATTACTGTAGAGCCTGGTGTTTTTTT |
| rs4680FA | A | TACGGTTATTCGGGCTCCTGTGATGGTGGATTTCGCTGTCA |
| rs4680FG | G | TTCCGCGTTCGGACTGATATGATGGTGGATTTCGCTGTCG |
| rs4680FP |  | TGAAGGACAAGGTGTGCATGCTTTTTTTTTTTT |
