# Supplementary material for: The association between serotonin-related gene polymorphisms and susceptibility and early sertraline response in patients with panic disorder
Source: BMC Psychiatry. 2020 Jul 28;20:388. doi: 10.1186/s12888-020-02790-y (PMC7388522; doi:10.1186/s12888-020-02790-y)
Supplement: Supplementary file 4 — Additional file 4: Table S4. Power of the study at current sample size [file 12888_2020_2790_MOESM4_ESM.docx]

Table S4. Power of the study at current sample size

| SNP | Type of test | MAF^#^ | Ψ**^*^** | Power | Required sample size  (cases vs controls) | Whether the present study met the sample size requirement (YES/NO) |
| --- | --- | --- | --- | --- | --- | --- |
| rs140701 | Allelic | 0.50 | 2.0 | 0.8 | 136 vs 136 | YES |
| rs3813034 | Allelic | 0.18 | 2.0 | 0.8 | 167 vs 167 | YES |
| 5-HTTLPR | Allelic | 0.13 | 2.0 | 0.8 | 141 vs 141 | YES |
| STin2 | Allelic | 0.15 | 2.0 | 0.8 | 207 vs 207 | YES |
| rs6295 | Allelic | 0.20 | 2.0 | 0.8 | 172 vs 172 | YES |
| rs6313 | Allelic | 0.41 | 2.0 | 0.8 | 132 vs 132 | YES |
| rs4680 | Allelic | 0.32 | 2.0 | 0.8 | 138 vs 138 | YES |

#:The minor allele frequency in Chinees Han population from 1000Genome (https://www.internationalgenome.org/). **^*^**: The odds ratio of exposure in cases relative to controls.
