# Supplementary material for: The association between serotonin-related gene polymorphisms and susceptibility and early sertraline response in patients with panic disorder
Source: BMC Psychiatry. 2020 Jul 28;20:388. doi: 10.1186/s12888-020-02790-y (PMC7388522; doi:10.1186/s12888-020-02790-y)
Supplement: Supplementary file 5 — Additional file 5: Figure S1. PD patients recruit flow chart. [file 12888_2020_2790_MOESM5_ESM.doc]

Patients who met the DSM-IV diagnostic criteria for PD (n=322)

Patients who agreed for this study (n=311)

Patients who refused for this study (n=11)

Patients who excluded due to comorbid somatic disorders or other reasons(n=24)

Patients who joined and interviewed by SCID-1in this study (n=287)

Patients who excluded due to comorbid other psychiatric disorders (n=42)

Patients who were eligible (n=245)

PD patients were eventually recruited (n=233)

Patients were lost follow-up (n=12)

Figure S1. PD patients recruit flow chart.
